# Is There a Major Role for Undetected Autism Spectrum Disorder with Childhood Trauma in a Patient with a Diagnosis of Bipolar Disorder, Self-Injuring, and Multiple Comorbidities?

**DOI:** 10.1155/2019/4703795

**Published:** 2019-05-26

**Authors:** Claudia Carmassi, Carlo Antonio Bertelloni, Gianluca Salarpi, Elisa Diadema, Maria Teresa Avella, Valerio Dell'Oste, Liliana Dell'Osso

**Affiliations:** Department of Clinical and Experimental Medicine, University of Pisa, Pisa, Italy

## Abstract

This case report highlights the relevance of the consequences of trauma in a female patient with an undetected autism spectrum disorder (ASD) affected by bipolar disorder (BD) with multiple comorbidities. A 35-year-old woman with BD type II, binge eating disorder and panic disorder was admitted in the Inpatient Unit of the Psychiatric Clinic of the University of Pisa because of a recrudescence of depressive symptomatology, associated with increase of anxiety, noticeable ruminations, significant alteration in neurovegetative pattern, and serious suicide ideation. During the hospitalization, a diagnosis of ASD emerged besides a history of childhood trauma and affective dysregulation, marked impulsivity, feeling of emptiness, and self-harm behavior. The patient was assessed by the Autism-Spectrum Quotient (AQ), Ritvo Autism and Asperger Diagnostic Scale (RAADS-R), the Adult Autism Subthreshold Spectrum (AdAS Spectrum), Trauma and Loss Spectrum (TALS-SR), and Ruminative Response Scale (RRS). Total scores of 38/50 in the AQ, 146/240 in the RAADS-R, 99/160 in the AdAS Spectrum emerged, compatible with ASD, 47/116 in the TALS-SR, and 64/88 in the RRS. We discuss the implications of the trauma she underwent during her childhood, in the sense that caused a complex posttraumatic disorder, a lifelong disease favored and boosted by the rumination tendency of high functioning ASD.

## 1. Introduction

The fifth edition of the Diagnostic and Statistical Manual of Mental Disorders (DSM-5) defines autism spectrum disorder (ASD) on the basis of two major criteria: (A) persistent deficit in communication and social interaction and (B) narrow interests and repetitive behaviors. Diagnosis has also to be integrated with a specifier stating whether the patient is or not* with language or intellectual impairment* [1].

ASD prevalence varies across studies from 0.76% to 2.64% in the general population, while prevalence rates in adult psychiatric inpatients vary from 2.4% to 9.9% [2]. All studies agree on the fact that prevalence has increased during the last 20 years, most probably because of a better recognition of the condition by physicians [3].

In this regard, it is important to recall the possibility that patients may present normal intelligent quotient and no verbal deficit that had been recognized to lead to a delay in the diagnosis, with a tendency of appropiate treatments to be postponed more frequently than other types of ASD [4, 5]. Several epidemiological and clinical studies have also found four time higher rates of ASD in males with respect to females [6], but some authors have speculated that there could be a diagnostic bias based on gender-specific adaptation skills that allow female patients to hide their social difficulties [7]. In this regard, most recent researches have also suggested the concept of autistic traits in order to define milder manifestations, qualitatively similar to the characteristic features of ASD, such as subthreshold social and communication deficits, unusual personality features, and stereotyped behaviors, continuously distributed in the general population and frequently found among unaffected relatives of people with autistic conditions [8].

Subjects with ASD commonly present a wide range of comorbidities: not only medical conditions (such as epilepsy, gastrointestinal problems, and immune dysregulation) but also psychiatric disorders, particularly anxiety, and depression with prevalence rates of 42-56% and 12-70%, respectively [9]. There is also evidence for comorbidity with obsessive-compulsive, psychotic, substance use, oppositional defiant, eating and personality disorders, especially in high functioning subjects with ASD [9–13]. Interestingly, despite increasing evidence is highlighting high rates of trauma exposure and possible vulnerability to posttraumatic stress symptoms, few studies searched for the prevalence of posttraumatic stress disorder (PTSD) among patients with ASD, reporting low rates [14].

In this paper, we present the case of a patient with bipolar disorder, binge eating disorder, self-harm behaviors, impulsivity, affective dysregulation, self-devaluation, and feeling of emptiness and instability in the relationships, hospitalized at the Inpatient Unit of the Psychiatric Clinic of the University of Pisa, in which an undetected diagnosis of ASD, without language or intellectual impairment, with childhood trauma emerged. This suggested a possible major role of these conditions as a vulnerability factor for the following development of severe multiple psychopathologies.

## 2. Case Presentation

### 2.1. Clinical Details

The patient (XX), a 35-year-old woman, was admitted to the Inpatient Unit of the Psychiatric Clinic of the University of Pisa for a major depressive episode. She was not married, was unemployed despite her educational achievement, and lived alone in her own house receiving an invalidity pension. She reported a family history of psychiatric disorders (a brother with panic disorder). XX was afflicted by multiple medical comorbidities, such as obstructive sleep apnea syndrome, polycystic ovary syndrome, hypertension, irritable bowel syndrome, and severe obesity (Body Mass Index was 39).

At the time of hospitalization, she reported low mood, abulia, decreased energy, apathy, anhedonia, feelings of sadness and inadequacy, and severe thoughts of death with suicide plans. She reported herself to be very anxious, tense, and irritable, with panic attacks (intense fear, palpitation, shaking, sweating, and sensation of smothering), and referred to staying at home all day because the streets smelled badly and noises were too unbearable to be sustained. Eating and sleep behavior patterns were totally disrupted. The clinical and diagnostic evaluation revealed also how XX presented narrow and unusual interests (particularly numbers and statistic and horror movies), strict adherence to her peculiar routine, difficulties to begin or carry on relationships, cognitive inflexibility, hyperreactivity to sounds, tastes, and lights, affective dysregulation, self-harm behaviors, marked impulsivity, and feeling of emptiness. All these symptoms were associated with low adaptation and social withdrawal. When inserted in a social context, she often put on big headphones to isolate herself and avoid noises.

Born in Ecuador, XX referred feelings of social incompetence, marked anxiety, excessive adherence to routines, rigidity of thinking, and distress to daily life change since she was a child. Since late childhood, she also reported severe weight instability with binge eating episodes associated with excessive and rigid food selection. At the age of 20, she had moved from Ecuador to a city in south Italy, where she attended a humanities faculty and at the age of 22 she reported her first full blown mood episode with mood deflection, abulia, apathy, anhedonia, irritability, marked ruminations, thoughts of death, progressive impairment in her adherence to sameness, and tendency to social isolation. All this symptomatology diminished without treatments within some months. In the same period, her eating behaviors got worse with an increase of binge episodes without compensatory behaviors and, consequently, progressive weight gain. At the age of 23, after the achievement of the university degree, she moved to another European country, where she attended an MSc program in Astrophysics, developing just a few months later a new depressive episode, complicated by self-harming behaviors (she began to cut herself on legs and arms) in order to face sadness, emotional pain, and other negative emotions. For these symptoms, she first contacted a psychiatrist and was treated with citalopram (up to 20 mg/day). At the age of 25 years, she suspended the pharmacological treatment starting a cognitive-behavioral psychotherapy focused on her social and eating behavioral difficulties, which she continued until the time of the current hospitalization. From the age of 27 to the age of 31 years, she reported progressive worsening of the mood symptoms and global functioning despite various treatments with SSRIs (escitalopram, paroxetine, and fluoxetine), followed by a hypomanic episode, for which she was started on mood stabilizers (carbamazepine). At the age of 31, she graduated in the MSc in Astrophysics and returned to a city of central Italy, reporting after a few months later a new severe depressive episode, associated with daily self-cutting behavior and suicide ideation with need of the hospitalization at the Psychiatric Clinic of the University of Pisa.

XX was admitted with diagnosis of bipolar disorder comorbid with anxiety and eating disorder.

During her hospitalization, XX was also administered the following assessment instruments on the basis of her personality and symptomatology: the Structured Clinical Interview for DSM-5 Disorders (SCID-5), the Autism-Spectrum Quotient (AQ) [15], the Ritvo Autism and Asperger Diagnostic Scale (RAADS-R) [16], the Adult Autism Subthreshold Spectrum (AdAS Spectrum) [17], the Trauma and Loss Spectrum (TALS-SR) [18], and the Ruminative Response Scale (RRS), reviewed and translated in Italian [19, 20].

By means of the SCID-5, a diagnosis of bipolar disorder type II, comorbid with panic disorder and binge eating disorder, emerged. Further, a first diagnosis of autism spectrum disorder without intellectual disability also resulted by the assessment instruments. A history of repeated sexual abuses during her childhood by a familiar member also emerged. During her hospitalization, XX received psychopharmacological treatment firstly with mood stabilizer (lithium 600 mg), antidepressants (amitriptyline 25 mg), and antipsychotic (perphenazine 2 mg). Upon diagnostic evaluation reported, we changed amitriptyline with sertraline 50 mg and perphenazine with aripiprazole 5 mg, showing a significant clinical global improvement. Furthermore, after discharge from the hospitalization, the patient resumed a cognitive-behavioral psychotherapy focused on traumatic process elaboration.

### 2.2. Assessment Instruments

The patient fulfilled the following scales and gave written informed consent to the processing of health data for research purposes.

The AQ is a self-report measure of autistic traits in patients from normal to high IQ. It is constituted by 50 questions, grouped in five different areas:* social skill, attention switching, attention to detail, communication, *and* imagination*.

The RAADS-R is the revised version of the Ritvo Autism Asperger's Diagnostic Scale [21] and includes four domains (*social relatedness, circumscribed interests, language and communication, and sensorimotor and stereotypies*).

The AdAS Spectrum is part of the “Spectrum Project” and it is conceived to identify the lifetime symptomatology of the entire autism spectrum, from subthreshold traits to the symptoms that fulfill the diagnostic criterion. It includes 160 items grouped in seven domains:* childhood/adolescence, verbal communication, nonverbal communication, empathy, inflexibility and adherence to routine, restricted interests and rumination, and hyper- and hyporeactivity to sensory input*.

The TALS-SR is a self-report measure of lifetime stress related spectrum. It includes 116 items, grouped in 9 domains:* loss events, grief reactions, potentially traumatic events, reactions to losses or upsetting events, reexperiencing, avoidance and numbing, maladaptive coping, arousal, and personal characteristics/risk factors*.

The RRS is an instrument to quantify the tendency to ruminative thinking of the patient; it is composed by 22 items that inspect 3 dimensions:* depression* thoughts,* brooding*, and* reflection*.

The patient reported a total of 38 over 50 at the AQ with the following scores at its domains:* social skill* (10 over 10),* attention switching* (9 over 10),* communication* (9 over 10),* attention to detail* (5 over 10), and* imagination* (5 over 10). She also showed a total score of 146 over 240 at the RAADS-R with these succeeding domains scores:* social relatedness* (74 over 117),* circumscribed interests* (27 over 42),* language and communication* (12 over 21), and* sensorimotor and stereotypies* (33 over 60). XX reported 99 over 160 at the AdAS Spectrum with the highest score in the* nonverbal communication* (20 over 28),* inflexibility and adherence to routine* (22 over 43),* restricted interests and rumination* (17 over 21), and* hyper-/hyporeactivity to sensory input* (12 over 17) domains, followed by* childhood/adolescence* (12 over 21),* verbal communication* (12 over 18), and* empathy* (4 over 12) domains. XX reported 47 positive items over 116 at the TALS-SR. In particular, she reported the following traumatic events: unwanted sexual advances and physical or sexual abuse, also corroborated by the endorsement of being victim of a crime, besides changes in homes, caregivers, schools, jobs; painful break-up of relations; divorce in the family; loss or death of a cherished pet; repeated failure in school or at work; and serious accident or injury. Further, she endorsed the following scores at TALS-SR domains:* loss events* (4 over 10),* grief reactions* (11 over 27),* potentially traumatic events* (8 over 21),* reaction to losses or upsetting events* (10 over 18),* reexperiencing* (4 over 9),* avoidance and numbing* (3 over 12),* maladaptive coping* (4 over 8),* arousal* (1 over 5), and* personal characteristics*/*risk factors* (2 over 6). She reported 64 over 88 at the RRS:* brooding* (28 over 44),* depression* (20 over 24), and* reflection* (16 over 20).

## 3. Discussion

We present the case of XX, a 35-year-old woman with a diagnosis of bipolar disorder type II, binge eating disorder, panic disorder, and an undetected diagnosis of ASD without language or intellectual impairment. XX also showed marked ruminations and anxiety symptoms with somatizations as well as symptoms of cluster B personality disorder, such as affective dysregulation, marked impulsivity, feeling of emptiness, self-harm behavior, and instability in relationships.

Despite her high educational achievements, she presented poor social skills, cognitive inflexibility, and hyperreactivity to sensory stimulation with a severe impairment in work and social functioning. In this regard, she reported scores at the RAADS-R (154) and the AQ (38), compatible with a diagnosis of ASD as well as high scores in most of the AdAS Spectrum domains, particularly in* restricted interests/ruminations, hyper-/hyporeactivity to sensory input, and nonverbal communication *and* verbal communication *domains.

XX reported at the TALS-SR sexual abuses during childhood with lifetime posttraumatic stress spectrum symptoms, particularly* reexperiencing* and* maladaptive coping*.

Most recent literature on ASD female suggests adaptive coping strategies to be dramatically impaired compared to typical neurodeveloped individuals when facing traumatic experiences, particularly sexual abuse [22]: these elements are remarkable risk factors for the onset of PTSD. In parallel, there is a great amount of data in literature suggesting autistic women to be at higher risk of sexual harassment because of their difficulties in understanding other people intentions [23, 24].

Some authors have suggested ruminations to be the link between ASD and PTSD [25–30]: the cognitive processes of ASD patients often involve rumination, a sort of repetitive and inconclusive thought, that has been associated with anxiety, depressive mood, and a higher risk to develop psychopathological consequences when exposed to trauma, since this type of approach may prevent correct processing of traumatic experiences and it could represent a major risk factor for PTSD [28–32]. Recent studies showed correlations between autistic traits and mood spectrum disorders, both direct and mediated by ruminations and posttraumatic stress symptoms [33, 34]. Rumination indeed maintains and augments depressive symptomatology [35], so it can be interpreted as a way to avoid the processing of negative emotion that does not help to deal with the mood disorder [36].

Concerning the comorbidity between ASD and posttraumatic stress symptomatology, we must face two main issues. First, ASD could remain undiagnosed for long time as seen in this patient, while the person draws attention of the clinician only when other major psychiatric illnesses have arisen, for example, depression, anxiety, or suicidal behaviors [37–39]. Second, traumatic experiences too could be underdiagnosed in autistic patients, because of their low insight concerning traumatic events and the related difficulties to report them during anamnesis. Indeed, autistic patients have all been reported to be generally more predisposed to undergo traumatic interpersonal experiences within social interactions, such as bullying at school, due to their deficits in communication and keeping context-suitable behavior [40]. King (2010) presented a similar prospective: high functioning autistic patients and people with autistic traits are constantly exposed to stressors because of daily social interactions and inevitable changes to routine, and they could develop a posttraumatic stress symptomatology defined by the International Classification of Diseases (ICD-11) as complex-PTSD (cPTSD) [41]. cPTSD encompasses PTSD criteria, besides some peculiarities: affective dysregulation, maladaptive behaviors, self-devaluation, and instability in relationships. These latter are essentially similar to the ones that characterize borderline personality disorder (BPD) [42] and often misdiagnosed with [41, 43]. In this regard, biologic and emotional reactions to traumatic events in patients with ASD are usually stronger and more dysregulated [44, 45], with cPTSD symptomatology that could lead to BPD (King, 2010): these elements could lead clinicians to diagnose BPD, instead of cPTSD, neglecting autistic traits in these patients. cPTSD and BPD etiopathogenetic and clinical features amply overlap; in fact both of them are characterized by affective dysregulation, maladaptive coping, self-devaluation, and instability in relationships [46–48]. A growing body of evidence is showing the high correlation between ASD traits and BPD, where some autistic features can be related to difficulties in social relationship and an increased suicidal ideation [8, 34, 49–53].

Eating disorders are connected to both autistic trait and PTSD: higher autistic traits are correlated to augmented prevalence of eating disorder [54]; alterations in eating behaviors are also frequently reported in patients with traumatic experiences [55], and there is a strong association between BED and PTSD [56, 57].

Regarding the pharmacological approach used on the patient, we combined a tailored therapy to treat the different psychopathologic domains, focusing on the patient pharmacologic history and her medical comorbidities: we chose lithium as the first-choice mood stabilizer in bipolar disorder [58–61], sertraline as antidepressant [62], and aripiprazole to act on behavioral disturbances of ASD [63, 64].

The emblematic case described in this report highlights the clinical relevance of investigating traumatic experiences and stress related symptomatology in patients with ASD or autistic traits. Further researches are needed to define the psychopathological pattern in subjects with trauma related symptoms and to provide a correct framework and a suitable treatment.
